# Symptoms mimicking dementia in a 60-year-old woman with bipolar disorder: a case report

**DOI:** 10.1186/1756-0500-7-381

**Published:** 2014-06-21

**Authors:** Froukje H Woudstra, Aida T van de Poel-Mustafayeva, Maya V van der Ploeg, Jeroen J de Vries, Rixt F Riemersma van der Lek, Gerbrand J Izaks

**Affiliations:** 1 University of Groningen, University Medical Centre Groningen, University Centre for Geriatric Medicine, Internal Postcode AA43, P.O. Box 30.001, Groningen 9700 RB, The Netherlands; 2 Geriatric Psychiatry Clinic, University of Groningen, University Medical Centre Groningen, University Centre for Psychiatry, Internal Postcode AA82, P.O. Box 30.001, Groningen 9700 RB, The Netherlands; 3 Department of Neurology, University of Groningen, University Medical Centre Groningen, Internal Postcode AB51, P.O. Box 30.001, Groningen 9700 RB, The Netherlands; 4 Mood Disorders Clinic, University of Groningen, University Medical Centre Groningen, University Centre for Psychiatry, Internal Postcode CC44, P.O. Box 30.001, Groningen 9700 RB, The Netherlands

**Keywords:** Elderly, Reversible dementia, Depressive disorder, Bipolar disorder, Neurodegenerative disease

## Abstract

**Background:**

Dementia is generally considered an irreversible process of cognitive decline that can be caused by different neurodegenerative diseases. However, in some cases, dementia is caused by a non-neurodegenerative disease, such as an affective disorder. In these cases, the dementia can be reversible. Nevertheless, cognitive symptoms due to an affective disorder are often difficult to distinguish from a depressed mood due to a neurodegenerative disease. Especially in elderly patients with a history of affective disorder, a potentially reversible cause can be missed.

**Case presentation:**

We describe a 60-year-old white woman with bipolar disorder, depressive symptoms, a movement disorder and severe cognitive impairment, in whom a neurodegenerative disease was seriously considered. She was referred to our clinic for further investigation because initial treatment of the depressive episode with antidepressants, mood stabilizers and electroconvulsive therapy (ECT) had not been successful. However, despite extensive evaluation, we could not find evidence for a neurodegenerative disease and the patient mostly recovered after discontinuation of different psychotropic medications and treatment with nortriptyline.

**Conclusions:**

Our case shows that improvement of severe cognitive impairment in individual cases is possible. In our opinion, this underlines the necessity of a careful re-evaluation of the patient’s symptoms at presentation and the course of the disease as well as a critical review of the prescribed medications.

## Background

Dementia is a syndrome of cognitive deficits that is generally characterised by impairment in short- and long-term memory, and is often associated with impairment in abstract thinking or judgment, other disturbances of higher cortical function such as aphasia or apraxia, or personality change [1]. The cognitive deficits are severe enough to interfere significantly with work or usual social activities or relationships with others, and are not only present during a delirium. Dementia is most commonly caused by a neurodegenerative disease, such as Alzheimer’s disease, vascular dementia or Parkinson’s disease [2]. Therefore, dementia is generally considered an irreversible process of cognitive decline.

Since more than a century, it has been known that dementia can also be caused by non-neurodegenerative diseases and in some cases, can even be reversible if the underlying cause is adequately treated. One of the firsts reports was about the successful treatment of dementia due to neurosyphilis [3], but (partially) reversible dementia can also be caused by several other diseases. Infections, metabolic disturbances, brain tumors, normal pressure hydrocephalus, subdural hematoma, and drugs are well known examples [4,5].

Among the most prevalent diseases that can cause reversible dementia are affective disorders. Affective disorders include major depressive disorder, bipolar disorder, dysthymic disorder and cyclothymic disorder [1]. In Europe, the lifetime (combined) prevalence of affective disorders is approximately 14%. Of these, a major depressive disorder is the most frequent with a lifetime prevalence of 12% and a 12-month prevalence of 4%. The 12-month prevalence of bipolar disorder accounts for 0.7%-1.1% [6-8]. These results have been confirmed in several studies.

It has been estimated that in approximately 1% of the cases, dementia is caused by a major depressive disorder [5]. Probably, this percentage is higher in persons aged <70 years. A study from the Rochester Epidemiology Project found that 3% of the patients aged <70 had dementia due to mental illness such as schizoaffective disorder and major depressive disorder [9]. It has also been suggested that in patients with dementia due to a major depressive disorder, the dementia may be completely reversible in 30% of the patients [4]. However, up till now, many questions remain unanswered. For example, little is known about the severity of cognitive deficits in patients with reversible dementia due to a depressive disorder, or whether the severity of these deficits determine the reversibility. Also, it is still unclear if reversibility of the cognitive deficits is dependent on the type of affective disorder. Although some studies report improvement of cognitive deficits after successful treatment of patients with a major depressive disorder with psychotic features [10,11], other studies report persisting cognitive deficits in patients with a bipolar disorder [12-14].

Bipolar disorders are generally divided into two groups. Patients with type 1 bipolar disorder have experienced at least one manic episode that may have been preceded or may be followed by one or more hypomanic or depressive episodes [15]. In contrast, patients with type 2 bipolar do not experience full manic episodes but have experienced at least one hypomanic episode in addition to one or more depressive episodes [15]. Between manic and depressive episodes, these patients are considered to be euthymic [15]. In the last decade, there has been a particular interest in cognitive deficits in patients with a bipolar disorder [12-14]. Several studies report a similar pattern and severity of cognitive deficits in manic and depressive episodes and euthymic phases [12,14]. Interestingly, however, cognitive deficits have been found to be related to the duration of the bipolar disorder and the total number of manic and depressive episodes [12,13].

The diagnosis of dementia has great implications for the patients and their relatives. Unfortunately, the diagnosis is often difficult to confirm in elderly patients with a history of affective disorder. Especially in these patients, there is a considerable risk of missing a potentially reversible condition such as a major depressive or bipolar disorder [16].

In this case study, we describe a patient with type 1 bipolar disorder, a dystonic movement disorder and severe cognitive impairment who met the diagnostic criteria for dementia. Eventually, however, the dementia appeared to be largely reversible. We conclude that it is highly likely that the dementia was the result of a depressive episode in combination with the use of psychotropic drugs.

## Case presentation

A 60-year-old white female patient was referred to our tertiary care hospital for a second opinion regarding a severe treatment-resistant depression and a dystonic movement disorder. She was first referred to the department of psychiatry. Because she also suffered from severe cognitive problems, she was subsequently referred to the department of neurology. After extensive neurologic evaluation she was referred back to the department of psychiatry.

**Figure 1:**
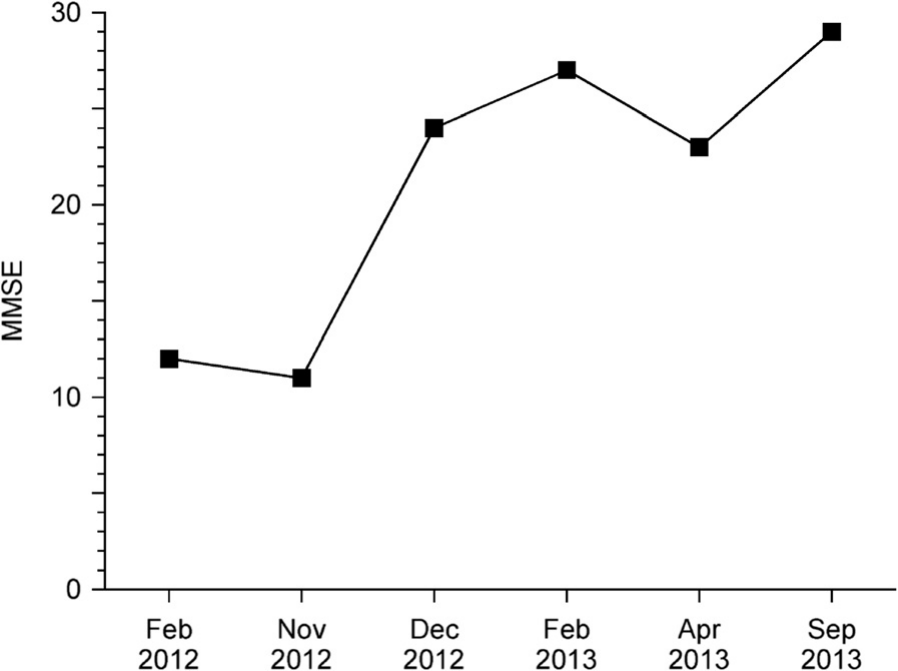
**Change in Mini-Mental State Examination score **[18]**during treatment.**

Her medical history included a type 1 bipolar disorder since the age of eighteen years, with frequent manic and depressive episodes and frequent admissions. Between manic and depressive episodes, she was functioning well and worked as a social worker. The last manic episode before the current episode was in 1992. After this episode, she had been stable on a combination of lithium and carbamazepine for eighteen years.In May 2010, the current disease episode started with manic symptoms. In that period, carbamazepine was discontinued because of a confirmed allergic reaction. It is possible that this event caused the manic episode. In December 2010, this was followed by severe depressive symptoms and psychosis. She was treated with different combinations of antipsychotics (quetiapine, risperidone, aripiprazole), mood stabilizers (lamotrigine, topiramate, valproic acid) and antidepressants (clomipramine), without any effect on symptoms. Lithium was discontinued after an emergency admission for lithium intoxication. In November 2011, the patient was treated with electroconvulsive therapy (ECT), also without improvement of the symptoms. During this period, she was admitted to a regional psychiatric hospital several times. In January 2012, her symptoms worsened and she developed a severe movement disorder with dyskinesia and frequent falling. These complaints improved after reduction of medication (zuclopentixol, clomipramine, lamotrigine, biperiden and quetiapine). In the last months before transfer to our hospital, her husband noticed progressive memory problems, especially in the short-time memory. In February 2012, the Mini-Mental State Examination (MMSE) score was 12 (Figure 1). Neuropsychological testing at that time was impossible because she was very disorientated and not cooperative.In October 2012, the patient was admitted to our tertiary hospital to further evaluate her depressive episode and cognitive problems. As common antidepressant treatments seemed to be ineffective and memory impairment progressed, we considered an underlying neurodegenerative disease as the main cause of her complaints. At that moment, she used lithium 500 mg a day and temazepam 10 mg a day as psychotropic medication. Furthermore, she used levothyroxin, nifedipine, nebivolol and alfacalcidol because of hypothyreoidism, hypertension and renal impairment, respectively. At admission, we saw a thin, anxious woman. Her consciousness was clear. Orientation in time and place was disturbed. She had difficulty with speech due to orofacial dyskinesia. Her MMSE score at that time was 11/30 points (Figure 1). During her stay, it was observed that she clearly had memory problems and could not find her way on the ward. She also had great difficulty in executive functioning tasks such as cooking and there were signs of apraxia. She was not able to dress and wash herself. In addition, she suffered from hallucinations and delusional ideas which were nihilistic in nature.

**Figure 2:**
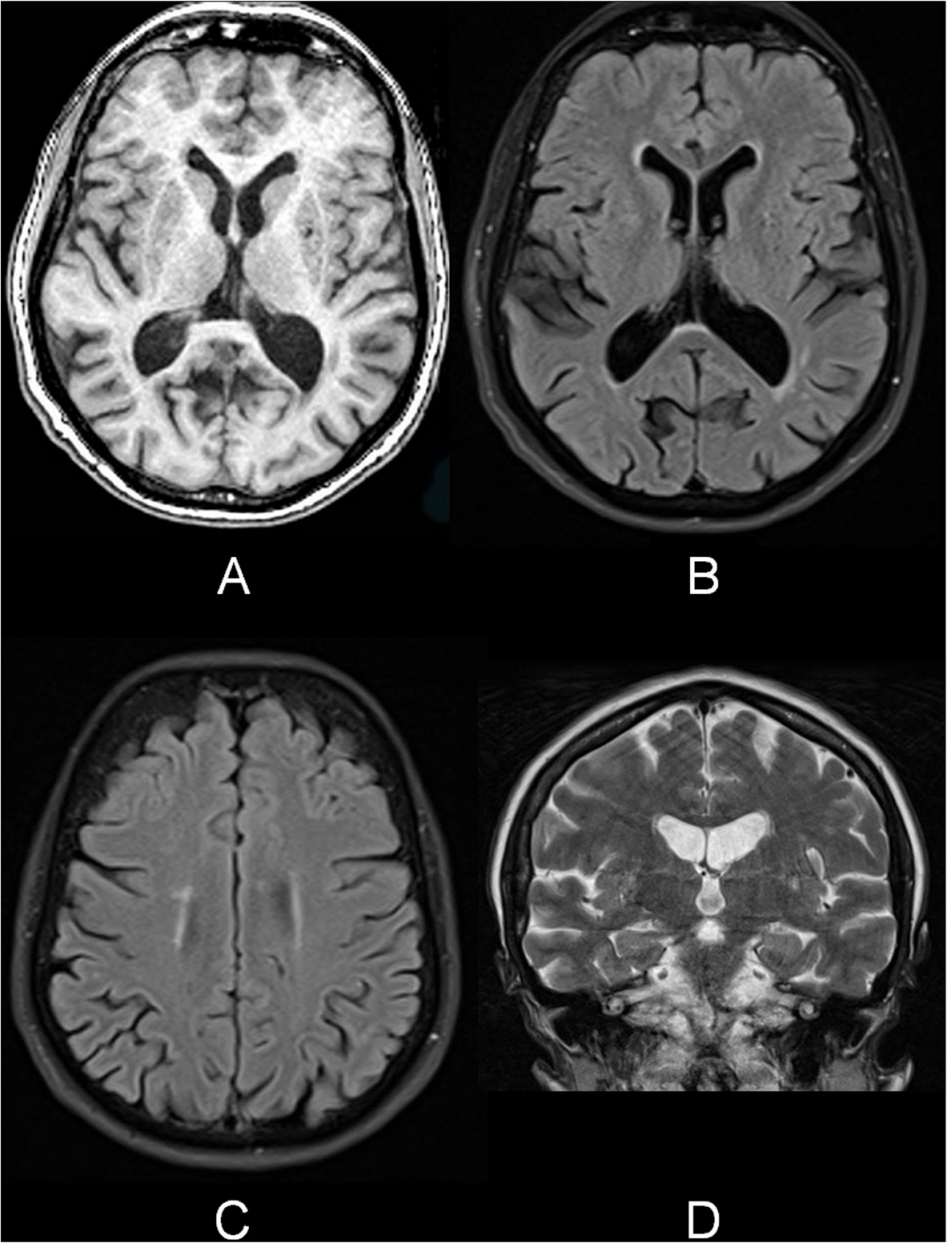
**Magnetic resonance imaging scan of the brain. A**, transverse T1-weighted image; **B** and **C**, transverse T2-weighted fluid attenuated inversion recovery image; **D**, coronal T2-weighted image of the medial temporal lobe and hippocampus. There was global cortical atrophy grade 1-2 with mild atrophy of the hippocampus (medial temporal lobe atrophy score, 1-2), on the left more pronounced than on the right. Compared to a magnetic resonance imaging scan performed one year earlier, there was no progression of these features. Furthermore, the T2-weighted images showed some punctuate subcortical hyperintensities but no other focal structural changes.

**Figure 3:**
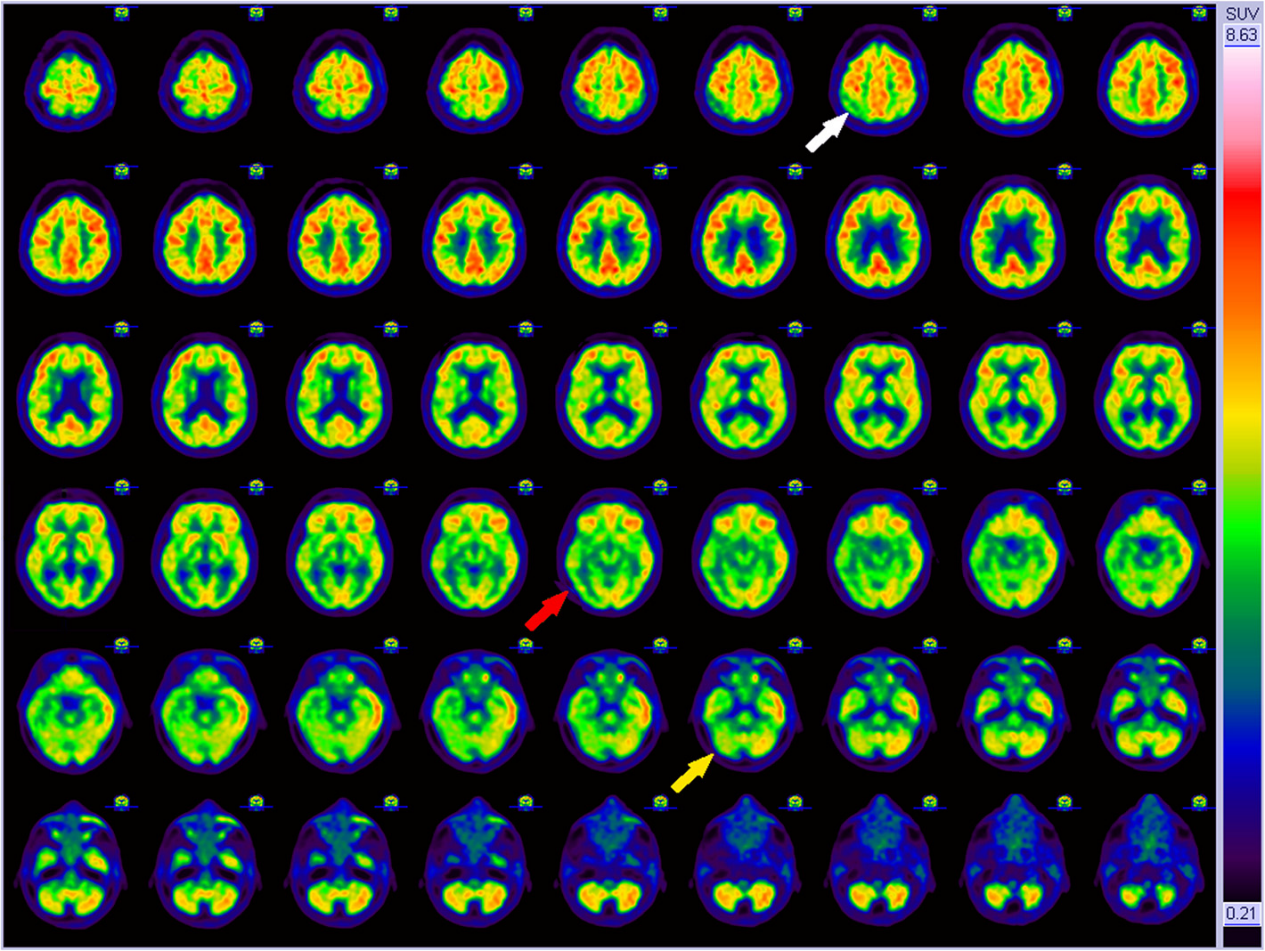
**18 F-fluoro-deoxy-glucose positron emission tomography scan of the brain (transverse images).** There was a non-specific pattern of hypometabolism of the right parietal cortex (white arrow) and temporal cortex (red arrow) as well as hypometabolism of the right cerebellar hemisphere (yellow arrow). The latter could not be explained by crossed cerebellar diaschisis as in that case the left cerebellar hemisphere would have shown a hypometabolic pattern.

The differential diagnosis included neurodegenerative disease or cognitive impairment as the result of severe depression with psychotic features. Because her situation was very severe we decided to perform additional diagnostic investigations for neurodegenerative disease such as neuroimaging and CSF analysis. However, the results of the magnetic resonance imaging (MRI) scan and 18 F-fluoro-deoxy-glucose positron emission tomography (FDG-PET) scan of the brain did not match classic patterns of known neurodegenerative causes (Figures 2 and 3). In combination with the negative results of the cerebrospinal fluid (CSF) analysis (Table 1), it seemed unlikely that her cognitive impairment was caused by a neurodegenerative disease.

**Table 1:** Results of relevant laboratory tests

**Laboratory test**	**Result**	**Reference value**
Leucocytes, ×10^9^/L	6.5	4.0-10.0
Hemoglobin, mmol/L	8.3	7.5-9.9
MCV, fL	91	80-96
Thrombocytes, ×10^9^/L	196	150-350
CRP, mg/L	<5	<5
Serum creatinine, umol/L	105	<90
Calcium, mmol/L	2.53	2.20-2.60
Total protein, g/L	68	60-80
Albumin, g/L	46	35-50
Glucose mmol/L	5.2	4.4-5.5
Folic acid, nmol/L	19	4.0-30.0
Vitamin B12, pmol/L	412	145-450
Thyroid-stimulating homone, mE/L	2,6	0.5-4.0
Lithium, mmol/L	0.66	0.6-1.2
CSF		
Protein, mg/L	250	290-670
Glucose, mmol/L	2.8	2.2-4.4
Erythrocytes, ×10^6^/L	0	absent
Leukocytes, ×10^6^/L	1	<5
Beta-amyloid, pg/mL	968	>500
Total-tau, pg/mL	180	0-350
Phosphorylated-tau, pg/mL	43	<85
Paraneoplastic antibodies	negative	negative

*Abbreviations:CRP*, C-reactive protein; *CSF*, Cerebrospinal fluid; *MCV*, Mean corpuscular volume.

We decided to treat the patient for a severe depressive episode with psychotic features. After careful evaluation of prior medication use, we started with nortriptylin with monitoring of the serum levels. In the following months, we saw a slow but significant improvement of her mood disorder and cognitive functioning. The MMSE score improved to 29 points (Figure 1). Also, the severe movement disorder improved to minimal orofacial dyskinesia. The patient was able to perform activities of daily living as well as cooking and gardening independently.

## Conclusions

In clinical practice, doctors often see elderly patients with depressive symptoms and cognitive impairment. Differentiating between a major depression with cognitive symptoms and early dementia is a great challenge [17]. Depression or feelings of sadness can be the first symptom of a neurodegenerative disease, whereas memory complaints and other cognitive impairments can be part of a depressive disorder. In the past, cognitive impairment due to depressive disorder was called pseudodementia and there is a widespread belief that it can be improved by antidepressive treatment. However, evidence is scarce and often of low quality, due to small numbers of patients and the lack of randomized trials.

Improvement of cognitive impairment following antidepressive treatment has been reported in the literature [18]. The review of Tielkes *et al.* and the meta analysis of Semkovska *et al.*, describe a statistical significant effect of ECT on cognitive performance [10,11]. However, the effects are small and only measured as change in MMSE score. In a retrospective study, a mean improvement of 1.62 points on the MMSE score was measured [19]. In several prospective studies, no significant difference on MMSE score was measured [20,21]. Unfortunately, all studies were relatively small and in some studies patients with dementia (MMSE <24) were excluded. Wagner *et al.* describe a patient with dementia and an MMSE score of 21 points, who suffered from a unipolar depression. After five ECT sessions, his MMSE score had improved to 28 points. However, cognitive impairment in this patient was relatively mild compared to our patient. Furthermore, to our knowledge, there is no evidence of improvement in MMSE performance with other treatments than ECT.

Our patient had more severe cognitive impairment than any other patient described in the literature. Usually, such severe symptoms are caused by a neurodegenerative disease. However, despite extensive evaluation, we could not find any other evidence that supported the diagnosis of a neurodegenerative disease. The patient improved solely on a low dose of nortriptyline in addition to the restart of lithium and discontinuation of antipsychotic medication which is highly unlikely in case of a neurodegenerative disease. Although she did not completely improve to her premorbid level of functioning, improvement was striking and of great value for the patient and her family.

We assume that the severe cognitive symptoms at admission, were a combination of major depressive disorder with psychotic features, and the adverse effects of medication (mainly antipsychotics). The remaining cognitive deficits could be explained in the light of her long existent bipolar disorder with frequent manic and depressive episodes in the past and the long duration of her last episode. This has been previously described in the literature [12-14,22,23]. There is little evidence that lithium causes cognitive impairment [24].

The prominent movement disorder was probably drug induced dyskinesia and akathasia [25]. These complaints diminished after discontinuation of antipsychotic drugs. Although these movement disorders are well known side effects of antipsychotic drugs, they can also be a symptom in several neurodegenerative diseases. Therefore, we advocate that in the evaluation of movement disorders and neurodegenerative diseases, medication is carefully reviewed and stopped if possible.

Although convincing evidence regarding improvement of dementia due to depression is scarce, our case shows the remarkable recovery of severe cognitive impairment in a depressed 60-year-old woman. Although it took several months before her depressive and cognitive symptoms improved, the treatment of her symptoms was relatively simple with little to no side effects. In our opinion, this proves that improvement in individual cases is possible and underlines the necessity of careful re-evaluation of medical history and medication use. Without this re-evaluation, there is a considerable risk of missing a potentially reversible condition with great implications for the patient and his or her relatives.

Finally, in the fifth edition of the Diagnostic and Statistical Manual of Mental Disorders (DSM-5) that was recently published by the American Psychiatric Association (APA), the term dementia was replaced with the term major neurocognitive disorder [15]. We think that our case study illustrates the advantages of this new term. Although physicians and patients may be accustomed to the term dementia, dementia is generally associated with neurodegenerative and cerebrovascular diseases that have a progressive course and cannot be reversed or cured. As a consequence, the term reversible dementia easily leads to confusion. Furthermore, a considerable number of persons aged <70 years are diagnosed with dementia that is caused by nondegenerative, nonvascular diseases [9]. Therefore, in our opinion, major neurocognitive disorder is the preferred term used when referring to persons aged <70 years with severe cognitive deficits that are caused by nondegenerative, nonvascular diseases and may be amenable to curative treatment.

## Consent

Written informed consent was obtained from the patient for publication of this Case Report and any accompanying images. A copy of the written consent is available for review by the Editor-in-Chief of this journal.

## Abbreviations

CRP: C-reactive protein; CSF: Cerebrospinal fluid; ECT: Electroconvulsive therapy; FDG-PET: Fluorodeoxyglucose positron emission tomography; FLAIR: Fluid attenuated inversion recovery (MRI); MCV: Mean corpuscular volume; MMSE: Mini-Mental State Examination; MRI: Magnetic resonance imaging.

## Competing interests

The authors declare that they have no competing interests.

## Authors’ contributions

FW and MP treated the patient and provided follow-up care. AP supervised the inpatient treatment and hospitalization. JV reviewed the neurological assessment and interpreted the results of neuroimaging and spinal fluid. FW, GI and RR drafted the manuscript. All authors reviewed the manuscript for intellectual content. All authors read and approved the final manuscript.
